# Altered Functional Connectivity in Resting State Networks in Tourette’s Disorder

**DOI:** 10.3389/fnhum.2018.00363

**Published:** 2018-09-18

**Authors:** Siyan Fan, Odile A. van den Heuvel, Danielle C. Cath, Stella J. de Wit, Chris Vriend, Dick J. Veltman, Ysbrand D. van der Werf

**Affiliations:** ^1^Division of Social and Behavioural Science, Utrecht University, Utrecht, Netherlands; ^2^Department of Anatomy & Neurosciences, Amsterdam Neuroscience, Amsterdam UMC, Vrije Universiteit Amsterdam, Amsterdam, Netherlands; ^3^Department of Psychiatry, Amsterdam Neuroscience, Amsterdam UMC, Vrije Universiteit Amsterdam, Amsterdam, Netherlands; ^4^The OCD Team, Haukeland University Hospital, Bergen, Norway; ^5^Department of Psychiatry and Rob Giel Research Center (RGOC), University Medical Center Groningen, University of Groningen, Groningen, Netherlands

**Keywords:** resting-Ss, Tourette Syndrome, functional connectivity (FC), default mode network (DMN), frontalparietal network

## Abstract

**Introduction:** Brain regions are anatomically and functionally interconnected in order to facilitate important functions like cognition and movement. It remains incompletely understood how brain connectivity contributes to the pathophysiology of Tourette’s disorder (TD). By using resting-state functional MRI, we aimed to identify alterations in the default mode network (DMN), frontal-parietal network (FPN), sensori-motor network (SMN), and salience network (SN) in TD compared with healthy control (HC) subjects.

**Method:** In 23 adult TD patients and 22 HC, 3T-MRI resting-state scans were obtained. Independent component analysis was performed comparing TD and HC to investigate connectivity patterns within and between resting-state networks.

**Results:** TD patients showed higher involvement of the dorsal medial prefrontal cortex in the connectivity of the DMN and less involvement of the inferior parietal cortex in the connectivity of the FPN when compared to HC. Moreover, TD patients showed a stronger coupling between DMN and left FPN than HC. Finally, in TD patients, functional connectivity within DMN correlated negatively with tic severity.

**Conclusion:** We tentatively interpret the increased functional connectivity within DMN in TD patients as compensatory to the lower functional connectivity within left FPN. The stronger coupling between DMN and left FPN, together with the finding that higher DMN intrinsic connectivity is associated with lower tic severity would indicate that DMN is recruited to exert motor inhibition.

## Introduction

Tourette’s disorder (TD) is a neuropsychiatric disorder characterized by involuntary, repetitive and compulsive movements, expressed as motor, phonic, and complex tics (Freeman, 2013). The neurobiological model of TD suggests a key role for the frontal cortico-striatal-thalamo-cortical (CSTC) circuits (Leckman, 2002; Milad and Rauch, 2012; Ji et al., 2016). These CSTC circuits originate from specific regions of the frontal cortex and project to the basal ganglia via direct or indirect pathways, culminating in the thalamus before projecting back to the frontal regions (Alexander et al., 1986). In recent years, this model has been extended to include interactions at the cortical level, specifically involving the fronto-parietal network (FPN), based on emerging functional and structural neuroimaging evidence for its relevance to the pathophysiology of TD (Ganos et al., 2013; Worbe et al., 2015). Whereas the brain can be considered a sophisticated multi-network organ, anatomically and functionally interconnected in order to optimally accomplish, e.g., cognitive and motor functions (van den Heuvel and Hulshoff Pol, 2010), it is unclear whether CSTC circuit abnormalities in TD interfere with known resting-state networks.

Resting-state functional magnetic resonance imaging (rs-fMRI) is a popular neuroimaging method for measuring spontaneous slow blood-oxygen level-dependent (BOLD) fluctuations that show co-activation between brain regions during resting state (Biswal et al., 1997). Several resting-state networks have been identified that display high-level functional connectivity between widespread cortical and subcortical areas. Four of these networks are specifically relevant for TD.

The default mode network (DMN) consists of the posterior cingulate cortex (PCC)/precuneus, medial frontal and inferior parietal and temporal regions (Buckner and Vincent, 2007), and is thought to be at the core of integrating cognitive and emotional processes (Greicius et al., 2003), mind-wandering (Mason et al., 2007) and monitoring the world around us (Gusnard et al., 2001); abnormal functional connectivity in the DMN has been reported in movement disorders like Parkinson’s disease (Tessitore et al., 2012; Esposito et al., 2013) and Huntington’s disease (Wolf et al., 2012; Werner et al., 2014). Recently, Wen et al. (2018) studied the topological organization of functional connectivity networks in TD children by using graph theory measures. Compared to HC, pediatric TD patients showed altered topology of the DMN. Another study reported an intact DMN when adult TD patients were instructed to lie in the scanner and were allowed to tic (as comparing to no tic activity), although no direct comparison with HC was made (Neuner et al., 2014). Altered functional integrity of DMN has also been demonstrated in other neuropsychiatric disorders such as OCD (Stern et al., 2012) and ADHD (De La Fuente et al., 2013).

The fronto-parietal network, consists of the anterior prefrontal cortex, anterior cingulate cortex (ACC), anterior insula, anterior inferior parietal lobule and the caudate nucleus and likely underlies attention processing and higher order cognitive functions (Damoiseaux et al., 2006). The salience network (SN), including bilateral anterior insula, ACC, pre-SMA, amygdala, ventral striatum and substantia nigra, is also involved in attentional control, as well as responding to behaviorally salient events (e.g., error detection) and important for goal-directed behavior (Seeley et al., 2007; Ham et al., 2013). Church et al. (2009) investigated adolescent TD patients and used a region of interest (ROI) approach to examine functional connectivity in the FPN and the SN (cingulo-opercular network). In TD patients showed anomalous functional connections in both the FPN and the SN when compared to healthy controls (HCs). In adult TD patients, Worbe et al. (2012) quantified functional connectivity in CSTC circuits using global integration and graph theory measures. They showed overall more interactions among these anatomical regions and a global functional disorganization of all these networks in patients compared to HC (Worbe et al., 2012).

The sensorimotor network (SMN), connecting the precentral with postcentral gyri and the supplementary motor area (SMA) is involved in motor control (Damoiseaux et al., 2006). Although using different data analytical approaches, several recent studies have reported abnormalities in the SMN in TD patients. For instance, Ji et al. (2016) studied amplitude of low-frequency fluctuation (ALFF) and functional connectivity and showed abnormalities in the SMN regions in TD children compared to HC. A different study also reported on TD children, and showed altered intrinsic connectivity in the sensorimotor areas (Liao et al., 2017). Additionally, topological disruptions were also observed in the SMN in TD patients (Wen et al., 2018).

The main objective of the present study is to replicate and extend previous literature on brain functional connectivity in relation to the pathophysiology of TD in the four above-mentioned specific resting-state networks (DMN, FPN, SN, and SMN) by using a data-driven analytical approach – independent component analysis (ICA). We first aimed to identify alterations in functional connectivity within these networks in TD compared to HC. Second, we examined relationships between functional connectivity and disease severity in TD.

We hypothesized that altered functional connectivity would be observed in TD in the four targeted networks based on the previous literature; as these previous studies adopted different methodological approaches to quantify functional connectivity measures, we did not have a prediction on the direction of the alteration in our study. Moreover, we expected that any observed altered functional connectivity in TD would be associated with disease severity.

## Materials and Methods

### Participants

Twenty-three TD patients and 22 HC participated in the study. TD patients were recruited from the Dutch TD patient organization and the outpatient clinics of Altrecht Academic Anxiety Center. HC were recruited from a previously examined cohort (de Wit et al., 2012; de Vries et al., 2014; van Velzen et al., 2014). The Structured Clinical Interview for DSM-IV-TR (SCID-I) on axis I psychiatric disorders was used to screen all participants on in- and exclusion criteria (First et al., 2002). The diagnosis of TD was established by an expert psychiatrist, and a trained research assistant assessed disease severity using the Yale Global Tic Severity Scale (YGTSS) (Leckman et al., 1989). The Yale-Brown Obsessive Compulsive Scale (YBOCS, including both symptom list and severity scale) (Goodman et al., 1989) and the Obsessive-Compulsive Inventory-Revised (OCI-R) (Foa et al., 2002) were administered to assess obsessive-compulsive symptoms and severity in TD patients with comorbidity of obsessive-compulsive disorder (OCD). We used the Beck Depression Inventory (BDI) and Beck Anxiety Inventory (BAI) to assess participants’ mood and anxiety symptoms, respectively (Beck et al., 1988, 1996).

Patients were allowed to have psychiatric comorbidities (except for current or past psychotic disorder, addictions, or mental deficiency), as long as they had a primary diagnosis of TD. Furthermore, participants were excluded if they had a major neurological illness, MRI contra-indications, or insufficient command of the Dutch language. According to the SCID-I, 5 out of the 23 patients with primary diagnosis of TD also met criteria for (OCD). No HC had a current DSM-IV-TR axis-I disorder or a family history of TD. Nine TD patients currently used typical/atypical antipsychotic drugs. The current study was approved by the local ethical review board of the Amsterdam UMC, location VUmc, and all participants provided written informed consent.

### Behavioral Analyses

Demographic, clinical and behavioral measures were analyzed using IBM SPSS Statistics version 22 (IBM, Armonk, NY, United States), using two-sample *t*-tests (2-group comparison between TD patients and HC). Results were considered significant at *p* < 0.05, two-tailed. Non-parametric tests were used if data did not meet parametric assumptions.

### Image Acquisition

Imaging data were obtained from a 3-Tesla GE Discovery MR750 scanner (General Electric, Milwaukee, WI, United States) at the Amsterdam UMC, location VUmc equipped with an eight-channel phased-array head coil. Structural images were acquired using a three-dimensional T1-weighted sequence (256 × 256 matrix; voxel size = 1 mm × 0.977 mm × 0.977 mm; 172 sections). Two hundred functional images were acquired using a T2^∗^ -weighted single-shot gradient echo-planar imaging sequence (TR = 1,800 ms; TE = 35 ms; 64 × 64 matrix; field of view = 21.1 cm; flip angle = 80°) with 39 slices per volume (3.75 mm × 3.75 mm in-plane resolution; slice thickness = 3.0 mm; interslice gap = 0.3 mm) for whole-brain coverage.

### Functional Connectivity Analysis

#### Pre-processing

The Brain Extraction Tool (BET) from FSL 5.08^1^ (Smith, 2002) was first applied to remove non-brain tissue from the T1-weighted structural images. For the functional data, the first two volumes from the time-series per subject were removed in order to allow for magnetization saturation and enhance data sensitivity. We used Melodic 3.14 (FSL) for an ICA-based single-session denoising approach (Beckmann et al., 2005) using the following standard processing steps: high-pass filtering (100 s), motion correction with MCFLIRT (Jenkinson et al., 2002), voxel-wise demeaning and normalization of the voxel-wise variance, slice timing correction and spatial smoothing with high-pass filtering conducted using a Gaussian kernel of full width at half maximum (FWHM) = 5.0 mm. Average frame-wise displacement (FD) for each subject group was calculated to assess movement. Subjects were excluded if their movements exceeded 3 mm. Pre-processed and filtered functional data were co-registered with structural images by using FLIRT for optimization and registered into Montreal Neurological Institute (MNI) standard space in a two-step procedure, using 7 and 12 degrees of freedom, respectively. FSL FIX 1.061^2^, a method that auto-classifies ICA components as veridical or artifactual was applied to detect and remove noise from the time-series (i.e., noise components and motion confounds from the preprocessed dataset) for each subject. Prior to applying FIX, mean relative RMS (root mean squared) displacement (gross motion) from all subjects was examined.

#### Independent Component Analysis

**FIGURE 1:**
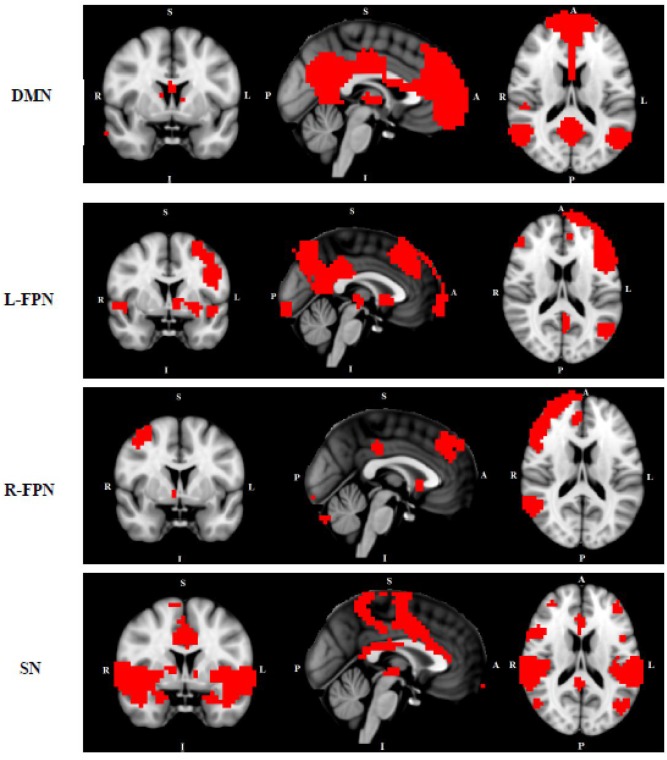
Networks of interest include the default mode network (DMN), left/right frontal-parietal network (L/R-FPN), salience network (SN) and sensori-motor network (SMN). Images thresholded at *Z* = 3.

We conducted multi-session temporal concatenation ICA on the denoized data sets, with dimensionality set to 20 (numbers of networks extracted from the analysis) to analyze ICAs at the group level. Five masks were created from the group ICAs with threshold *Z* = 3.0: (1) DMN, (2) left FPN, (3) right FPN, (4) SMN, and (5) SN (**Figure 1**). Dual regression analyses (Filippini et al., 2009) were performed to examine group differences between TD and HC; followed by permutation-based testing (5,000 permutations) using Randomize (Winkler et al., 2014), and Threshold-Free Cluster Enhancement (Smith and Nichols, 2009). A TFCE-corrected *p* < 0.05 was considered significant, and 0.05 < *p* < 0.09 was considered as a trend. Age and gender were controlled when added as covariates. Dual regression analyses were also conducted to access potential comorbidity effect [between “pure” (without comorbidity of OCD) and HC] and medication effect (between non-medicated TD and HC).

Within-group multiple regression analyses were conducted to investigate the relationship between the resting state network voxelwise strength and the patients’ tic severity (i.e., YGTSS).

### *Post hoc* Analysis on Inter-Network Functional Coupling

We followed up the main findings from the within-network functional connectivity analysis by investigating if the networks that showed between-group differences differed in their connectivity *between* networks. FSL’s dual regression was used to calculate subject-specific orthogonal time-series and spatial maps for DMN, left FPN and SN, which were defined by ICA (Filippini et al., 2009; Cole et al., 2010; Janes et al., 2012, 2014). We used the SN as a control network to establish the specificity of functional coupling between the DMN and the FPN. To examine functional coupling between the networks, we extracted the subject-specific time-series for each network per subject and calculated the correlation (Pearson’s r) of these time-series between the DMN and left the FPN, the DMN and the SN, the left FPN and the SN. The values were Fisher-z-transformed for use in further regression and correlational analyses. One-sample *t*-tests were first conducted on Fisher-Z coefficients across all participants to examine the presence and characteristics of functional coupling between each of the two networks (i.e., *t*-value is significantly positive = positive coupling; *t*-value is significantly negative = negative coupling; non-significant *t*-value = no coupling). This was followed up by independent samples *t*-tests for between-group comparisons on any identified functional coupling between networks (Putcha et al., 2015).

## Results

### Demographic and Clinical Characteristics

There was no difference of FD between TD (mean = 0.067 ± 0.050 mm) and HC (mean = 0.066 ± 0.021 mm). No subjects showed excessive movement (>3 mm) and there were no differences in movements between two subject groups, therefore all were included. Demographic and clinical characteristics of participants included in the between-group comparison (TD patients and HC) are shown in **Table 1**. No group differences were found on gender and educational level while on average the TD patients (mean age 34.6 ± 12.3 years) were younger than HC (mean age 43.3 ± 14.2 years). The patient group scored higher on TD severity, BDI and BAI than HC.

**Table 1:** Demographic and clinical measures of the study samples: including patients with Tourette’s disorder [with comorbidity of obsessive compulsive disorder (OCD)] and healthy controls.

	TD		HC		
	(*N* = 23)		(*N* = 22)		
Demographic measures	*N*	%	*N*	%	*p*
Gender (men)	15	65	14	64	0.59
	**Mean**	***SD***	**Mean**	***SD***	***p***
Age (years)	34.6	12.3	43.3	14.2	0.035
Education level^a^	8.1	1.7	8.2	2.0	0.74^b^
**Clinical measures**					
YGTSS (range 0–100)	46.3	16.7	N/A	N/A	
Y-BOCS severity (range 0–40)	16.8 (*N* = 5)	6.3	N/A	N/A
BDI	6.7	6.6	2.1	3.0	0.002^b^
BAI	7.8	7.3	2.1	2.8	<0.001^b^

^a^*Educational level was recorded in nine levels ranging from 1 (no finished education) to 9 (university training). ^*b*^Mann–Whitney *U* test. YGTSS, Yale Globe Tic Severity Scale; Y-BOCS, Yale-Brown Obsessive Compulsive Scale; BDI, Beck Depression Inventory; BAI, Beck Anxiety Inventory; TD, Tourette’s disorder; OCD, obsessive-compulsive disorder; HCs, healthy controls*.

### Functional Connectivity of Resting-State Networks

**FIGURE 2:**
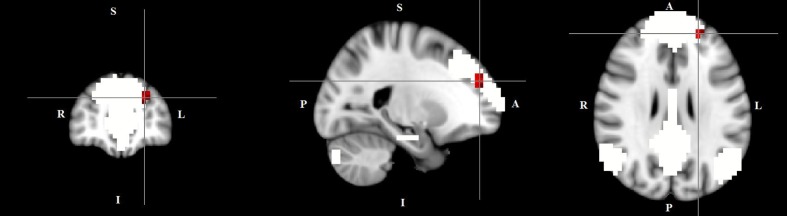
Higher functional connectivity in TD patients compared to HC in the default mode network. The image displays a higher involvement of the left dorsal medial prefrontal cortex region in the neural functional connectivity to the rest of the default mode network in TD patients (*X* = –22, *Y* = 49, *Z* = 28, *p* = 0.0056). For illustration purposes, *p* thresholded at 0.05.

**FIGURE 3:**
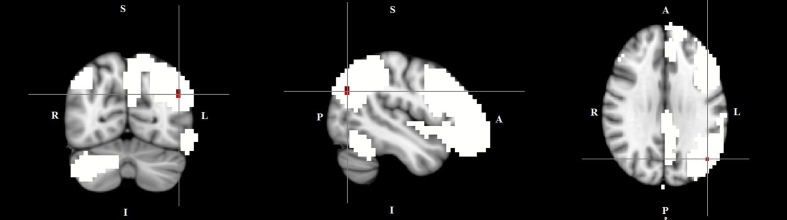
Lower functional connectivity in TD patients compared to HC in the left frontal-parietal network. The image displays a lower degree of involvement of the left inferior parietal cortex region in the neural functional connectivity to the rest of the left frontal-parietal network in TD patients (*X* = –46, *Y* = –69, *Z* = 29, *p* = 0.0186). For illustration purposes, *p* thresholded at 0.05.

The dual regression approach allows identifying which areas within a given network differ between TD patients and HC in terms of the strength of their contribution to the independent component, reflecting their level of connectivity to the rest of that network. We therefore refer to our findings as indices of functional connectivity in the below. This approach showed a significantly higher involvement of the left dorsal medial prefrontal cortex in the neural functional connectivity of the DMN in TD patients as compared to HC (*p* < 0.05) (**Figure 2** and **Table 2**). A significantly lower degree of involvement in the functional connectivity of the left FPN was also found in TD patients compared to HC at the left inferior parietal cortex (IPC) region (*p* < 0.05) to the rest of the network (**Figure 3** and **Table 2**). The above results remained significant when only “pure” TD patients (without comorbidity of OCD; *N* = 18) were compared to HC. A trend-higher functional connectivity in the DMN (from the same dmPFC region to rest of the DMN) was found when only the unmedicated TD patients (*N* = 13) were compared to HC (*p* = 0.09), while a significantly lower functional connectivity in the left FPN (from the same left IPC region to rest of the left FPN) remained. No differences were observed between TD and HC in any other targeted networks.

**Table 2:** Regions of significant difference between patients with Tourette’s disorder (TD) and healthy controls.

Two-group comparisons						
					
		Coordinates (MNI)				
**Resting-state networks**	**Region**	***X***	***y***	***z***	**Cluster size**	***p***

Default-mode network	Left dorsal medial prefrontal cortex	-22	49	28	38	0.0056
Left frontal-parietal network	Left inferior parietal cortex	-46	-69	29	20	0.0186

*The table includes all regions that met the Threshold-Free Cluster Enhancement significance level, corrected for *p* < 0.05. MNI, Montreal Neurological Institute*.

### *Post hoc* Analysis on Inter-Network Functional Coupling

**FIGURE 4:**
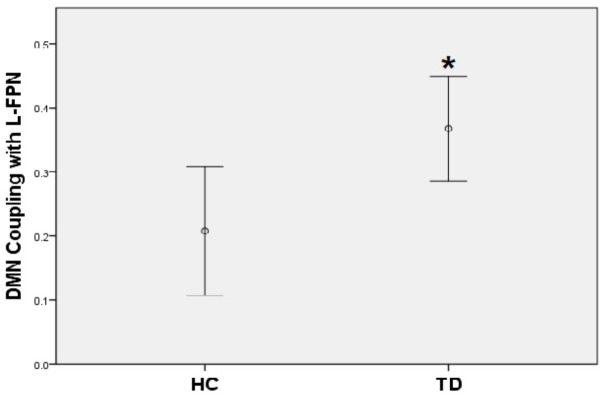
TD patients showed significantly higher positive coupling between default mode network and left frontal-parietal network compared to HC (^∗^*p* < 0.05).

A positive coupling between the DMN and the left FPN was revealed with the overall mean Fisher-Z coefficients across TD patients and HC showing statistical significance (Mean r square = 0.29, *SE* = 0.03), [*t*(44) = 8.78, *p* < 0.001]; while there was a significantly negative coupling between DMN and SN [Mean = -0.23, *SE* = 0.04, *t*(44) = -6.34, *p* < 0.001]. No coupling was found between the left FPN and the SN. When compared with controls, TD patients showed a stronger positive coupling between the DMN and the left FPN (*t* = -2.58, *p* = 0.01) (**Figure 4**).

### Correlations Between Functional Connectivity and Clinical Characteristics

**FIGURE 5:**
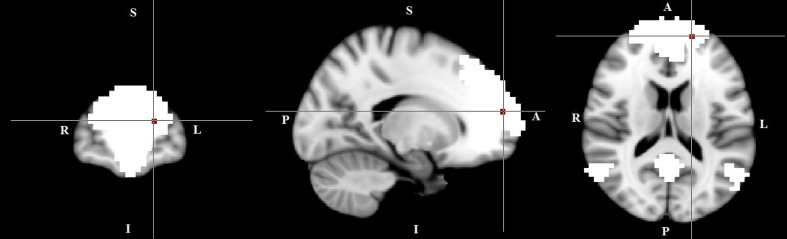
A negative correlation between functional connectivity in the default mode network and tic severity scores in TD patients (*X* = –17, *Y* = 59, *Z* = 16, *p* = 0.034). For illustration purposes, *p* was thresholded at 0.05.

In the within-group regression analyses (in TD patients only), functional connectivity in the DMN correlated negatively with tic severity (*p* < 0.05) (**Figure 5**).

## Discussion

The current study investigated functional connectivity in TD in four resting-state networks, i.e., the DMN, FPN, SMN, and SN. Within the DMN, TD patients compared to HC showed a higher connectivity between the left dmPFC and the rest of the network. Within the left FPN, TD patients compared to HC showed significantly lower connectivity between the left IPC region and the rest of the network. Also, TD patients compared with HC showed stronger coupling between the DMN and the left FPN. In TD, functional connectivity in the left dmPFC to rest of the DMN correlated negatively with tic severity.

Although the DMN finding appears to be at odds with Neuner et al. (2014), who reported ‘relatively preserved’ connectivity within the DMN in TD patients during free ‘ticcing’ during scanning, the two studies are not comparable due to different conditions. However, since our study subjects were instructed to lie still during scanning, we cannot rule out the possibility that the study findings may reflect an effect of tic suppression rather than a true resting-state in our TD patients. The dmPFC is suggested to be one of the highest associative centers in the frontal lobe participating in cognitive processes and autobiographical retrieval. In TD, thinner cortex of the dmPFC has been found to relate to more severe tic symptoms (Sowell et al., 2008), and lower white matter volume has also been demonstrated in this area (Greene et al., 2017). The dmPFC is a key node of the DMN, which is particularly relevant for processing highly demanding and complex cognitive tasks (Eickhoff et al., 2016). Although the DMN has been implicated in mind-wandering (Mason et al., 2007) and monitoring the world around us (Gusnard et al., 2001), it has also been linked to integrating cognitive processing (Greicius et al., 2003). Due to long-term struggling and coping with inappropriate acts, adult TD patients can achieve a variety of cognitive tasks by enhancing cognitive control (Mueller et al., 2006; Jackson et al., 2007). Therefore, although speculative at this stage, the increased dmPFC functional connectivity in the DMN might be associated with enhanced cognitive control as a compensatory cognitive mechanism developed by our adult TD patients as a result of long-term tic suppression.

Tourette’s disorder patients showed decreased functional connectivity in the left IPC within the FPN network compared to HC. The IPC is known for its role in attention processes, motor control and response inhibition (Rizzolatti et al., 1997; Rushworth et al., 2001; Cieslik et al., 2015; Fan et al., 2017). In TD, lower cortical thickness of the IPC was found to associate with more severe tic symptoms (Fan et al., 2017). Functionally, tic genesis corresponds to abnormal activation in the parietal cortex (the IPC and superior parietal cortex) (Stern et al., 2000; Bohlhalter et al., 2006), and higher IPC activity was found during successful inhibition in TD patients (Fan et al., 2017). The current study, although using a different methodological approach compared with other studies, shows results that are in line with two previous studies reporting abnormal functional connectivity within the FPN in adolescent and adult TD patients (Church et al., 2009; Worbe et al., 2012). The FPN is known for its role in attention processing (Biswal et al., 1995) and subserves attentional gating, shifting focus of interest (Dehaene and Naccache, 2001), and maintaining information content that is necessary for performance on online rapid adaptive control tasks (Dosenbach et al., 2008).

Anatomically, the FPN is closely connected with the DMN (Vincent et al., 2008) and the FPN co-activates with the DMN for production of internal thoughts (Smallwood et al., 2012). It has been proposed that the FPN serves as an attention control network that cooperates with the DMN to aid in either internal thought coordination or sustaining the internal thought stream and preventing it from disruption of external stimuli (Smallwood et al., 2012). In our follow-up inter-network connectivity analyses, findings were consistent with the suggestion of Smallwood et al. (2012), in which positive coupling between the DMN and the FPN across TD and HC was revealed. Additionally, we used the SN as a control network and investigated its functional coupling with both the DMN and the left FPN to further study whether this observed positive inter-network coupling was specific or a general feature of all four networks. The SN was negatively coupled with the DMN while no coupling was found between the SN and the FPN. Between-group comparisons showed that TD patients have significantly stronger coupling between the DMN and the FPN than HC.

Dysfunction of the FPN contributes to TD pathophysiology (Church et al., 2009; Worbe et al., 2012), possibly reflecting an inefficient ability of the FPN to prevent the disruption of internal thought streams that are generated by the DMN by external stimuli. Although speculative, one possible interpretation of our main findings might be that adult TD patients are able to boost their DMN functional connectivity. Such a suggestion seems to agree with our observed negative correlation between functional connectivity and tic severity in the DMN, such that a higher DMN activity is associated with a better tic suppression. It remains to be shown, nevertheless, whether the DMN plays an active role in the control of ticcing behavior.

No group differences between TD and HC were observed in the SMN and the SN. This is partly inconsistent with previous literature showing overall disorganizations in functional connectivity between cortical, basal ganglia and limbic regions in TD (Church et al., 2009; Werner et al., 2010; Worbe et al., 2012). We suspect that the absence of alterations in the SMN and the SN might reflect differences in methodological approaches used. Another explanation for the negative findings might be a lack of power of our study to identify potential differences between TD and controls because of the modest sample size in this study.

The current study has several additional limitations including: (1) difference in age between TD and HC groups; (2) the use of medication in some of our TD patients; (3) an *a priori* selection of networks of interest; (4); although each regression analysis was corrected for multiple comparisons using TFCE, we did not correct for the number of analyses in order to avoid false negative findings. Therefore, replication is needed in larger, independent and well-matched samples with sophisticated experimental designs to control for a possible tic-suppression effect in the future.

## Conclusion

In conclusion, compared with HCs, TD patients showed increased connectivity in the DMN and decreased connectivity in the left FPN, respectively, combined with a stronger positive functional coupling between these two resting-state networks. In addition, higher functional connectivity within the DMN was associated with a lower tic severity. Taken together, our results suggest that TD patients show enhanced functional coupling *between* the DMN and the FPN in order to compensate for altered functional connectivity *within* these two networks. This might be a compensatory mechanism to overcome motor tics.

## Author Contributions

SF contributed to data collection, research analyses, and manuscript written and edited. OH and DC contributed to study design and manuscript edited. SW contributed to manuscript edited. CV contributed to part of research analyses and manuscript edited. DV contributed to study design, part of research analyses, and manuscript edited. YW contributed to study design, part of research analyses, and manuscript edited.

## Conflict of Interest Statement

The authors declare that the research was conducted in the absence of any commercial or financial relationships that could be construed as a potential conflict of interest.

## Footnotes

1 https://fsl.fmrib.ox.ac.uk/fsl

2 https://fsl.fmrib.ox.ac.uk/fsl/fslwiki/FIX/
